# Enrichment of Probiotic Fermented Milk with Green Banana Pulp: Characterization Microbiological, Physicochemical and Sensory

**DOI:** 10.3390/nu10040427

**Published:** 2018-03-29

**Authors:** Carolina de Oliveira Vogado, Eliana dos Santos Leandro, Renata Puppin Zandonadi, Ernandes Rodrigues de Alencar, Verônica Cortez Ginani, Eduardo Yoshio Nakano, Sascha Habú, Priscila Araújo Aguiar

**Affiliations:** 1 Faculty of Health, Department of Nutrition, University of Brasília, Distrito Federal CEP 70910-900, Brazil; carol_vogado@yahoo.com.br (C.d.O.V.); renatapz@yahoo.com.br (R.P.Z.); vcginani@gmail.com (V.C.G.); priscilla01araujo@gmail.com (P.A.A.); 2 Faculty of Agronomy and Veterinary Medicine, University of Brasília, Distrito Federal CEP 70910-900, Brazil; ernandesalencar@unb.br; 3 Department of Statistic, University of Brasília, Distrito Federal CEP 70910-900, Brazil; eynakano@gmail.com; 4 Department of Environmental Technology, University Technological Federal of Paraná, Paraná CEP 80230-901, Brazil; sashabu@yahoo.com.br

**Keywords:** probiotic, green banana pulp, fermented milk, dairy

## Abstract

The aims of this study were (i) to evaluate the growth kinetic of *L. paracasei* LBC 81 in fermented milks enriched with green banana pulp (GBP); (ii) to evaluate the effect of the incorporation of GBP on the chemical composition and the sensory acceptance; and (iii) to study the viability of the probiotic and technological properties during refrigerated storage. The amount of GBP used were 3.0, 6.0 and 9.0 g/100 g. The results show that the higher the concentration of GBP added, the shorter the time taken to reach pH 4.6. It was observed that the incorporation of GBP did not affect negatively the viability of *L. paracasei* LBC 81 during storage. The fermented milk elaborated with 6.0 g/100 g of GBP was the most accepted. The present study indicates that the enrichment of fermented milk with GBP favors the stability of the probiotic strain, *L. paracasei* LBC 81 during storage.

## 1. Introduction

Probiotics are live microorganisms that are capable of colonizing the gastrointestinal tract (GIT) and, when consumed in adequate amounts, confer benefits to human health, such as prevention against some types of cancer, intestinal regulation, improvement of digestibility, reduction of lactose intolerance, reduction of side effects of antibiotics, and reduction of the symptoms of irritable bowel syndrome [1,2]. The lactic acid bacteria (LAB) group is composed of several probiotic strains. The *Lactobacillus* genus is the most representative of the probiotics [3] and it is widely used in foods.

Dairy products are the most important group of foods that carry probiotics, in which fermented milk is the most traditional group in this category. To obtain health benefits, it is recommended that a product contains at least 10^6^ Colony Forming Units (CFU)/g of the probiotic strain at the time of consumption [4]. Therefore, it is very important to study the growth and the viability of probiotic strains on food.

The viability of probiotic microorganisms is an important parameter in the development of probiotic foods, such as fermented milk. Several factors can affect the viability of probiotic strains in fermented milks, including their formulation ingredients [5], the presence of fruits pulp on formulation [6], the pH of the medium, water activity, oxygen content, and storage conditions of the product (i.e., temperature) [3].

Several ingredients have been added during the elaboration of yoghurts and fermented milks, with different objectives: to improve the nutritional value; to stimulate growth; and to accentuate the survival of the probiotic strain during the refrigerated storage period. Among these ingredients, studies have highlighted prebiotic substances (i.e., inulin) [7], quinoa flour [8] and fruit flours [9].

Another ingredient that has aroused interest in the elaboration of yogurts is green banana pulp [10,11]. The green banana is rich in several nutrients and bioactive compounds, such as resistant starch, phenolic acids, minerals and vitamins that are important to human health [12]. In addition to the health benefits of the consumer, it has been observed that the incorporation of green banana pulp does not affect the technological or sensory characteristics of the yoghurts.

The incorporation of green banana pulp (GBP) in the elaboration of yoghurt increases the growth of probiotic strains, such as *Lactobacillus acidophilus* and *Bifidobacterium bifidum* [10]. However, there are no related studies in the literature showing the effect of the incorporation of GBP on the growth kinetics of probiotic strains and in survival during refrigerated storage. Furthermore, it is important to evaluate the effect of the incorporation of GBP into other technological variables during storage, such as syneresis and color of the product.

To the best of our knowledge, there are no studies that have evaluated the effect of GBP on fermented milk characteristics. Furthermore, considering that GBP is a nutrient source that helps the growth of lactic bacteria, the characterization of the product regarding the survival of probiotic strains and technological parameters during refrigerated storage must be performed. Thus, the aims of this study were (i) to evaluate the growth kinetics of *L. paracasei* LBC 81 in fermented milks enriched with different amounts of green banana pulp; (ii) to evaluate the effect of the incorporation of green banana pulp on the chemical composition and the sensory acceptance of fermented milk; and (iii) to study the viability of the probiotic strain and the technological properties of fermented milks during refrigerated storage.

## 2. Materials and Methods

### 2.1. Microorganisms

The dehydrated culture of *Lactobacillus paracasei* subsp. *paracasei* LBC 81 was cultivated in a medium containing Reconstituted Skim Milk Powder (RSMP) (Nestlé, Araçatuba, São Paulo, Brazil) in an amount of 10 g/100 g to water weight. The culture of microorganism was immediately incubated in a bacteriological oven at 37 °C for 14 h. A second activation of the culture was performed under the same conditions. All the experiments in this study followed these culture activation steps.

### 2.2. Production of Base Formulation to Prepare Fermented Milks

Four base formulations were elaborated to produce the fermented milk with different amounts of GBP. The formulations’ bases were composed of 10.0 g/100 g RSMP and 7.0 g/100 g sugar in relation to the water weight associated with different amounts of GBP: (i) control sample—without addition of GBP; (ii) addition of 3.0 g/100 g of GBP; (iii) addition of 6.0 g/100 g of GBP and (iv) addition of 9.0 g/100 g of GBP. According to the manufacturer (La Pianezza^®^, Industrial District Bandeirants, Brazil), 100 g of GBP is composed of 20.00 g of carbohydrates, 1.17 g of proteins, 7.83 g of fiber, 1.17 mg of iron, 20.16 mg of magnesium, 0.50 mg of manganese, 235.00 mg of potassium, and it is free of total fats. The samples were homogenized with a sterilized mixer, distributed in glass jars, and immediately autoclaved at 121 °C for 15 min.

### 2.3. Production of Fermented Milks and Determination of Kinetic Parameters of the Fermentation

The autoclaved base formulation samples were cooled and inoculated with 10% of the activated culture of *L. paracasei* subsp. *paracasei* LBC 81. The inoculated milk was incubated at 37 °C for 14 h. After that, the kinetic parameters, pH and titratable acidity, were evaluated. pH was evaluated by a potentiometer (Digimed, modelo DM21, São Paulo, Brasil), and measures were made every two hours, until pH 4.6 or an equivalent value was reached. The titratable acidity was determined according to the methodology described by [13]; the results were expressed in g/100 g of lactic acid.

### 2.4. Chemical Composition

The chemical composition of the fermented milks enriched with GBP was determined by the evaluation of the moisture content [13]; the protein content was determined by the Kjeldahl method and ashes [14]; the crude fiber was evaluated using the Fiber Digester model MS444/CI (Marconi, Piracicaba, Brazil); and the total carbohydrate content present in the products was as the remainder—the values found for moisture, protein content, lipid content and ash content were subtracted from 100. The lipid content was measured using the fat extractor, Model ANKOM XT15 Extractor (Ankom Technology, New York, NY, USA) [15].

### 2.5. Sensory Evaluation

The sensory test for the fermented milk samples was performed with 113 panelists (age ranging from 18 to 58 years, 51% females and 49% males). The samples were randomized and monadically presented. The panelists evaluated five attributes: appearance, flavor, aroma, texture and overall acceptance, using a 9-point hedonic scale. The Free and Informed Consent Form was presented to the participants of the study and approved by the Research Ethics Committee (Parecer: 2.000.289) of the University of Brasilia.

### 2.6. Syneresis Measure in the Fermented Milks

The fermented milk syneresis was determined by the methodology described by Fiszman et al. [16] with adaptations. Flasks containing 10 mL of the fermented milk samples were rested at 4 °C, and at intervals of 3, 6, 9, 12, 18, 24 and 48 h, the serum released was collected with the aid of a pipette (20–200 μL). The syneresis was calculated as the ratio of the serum volume to the initial volume of the fermented milk samples and was expressed in mL/100 mL.

### 2.7. Characterization of the Fermented Milks during the Storage Period

The viability of the probiotics and the technological properties of the fermented milks enriched with GBP were evaluated in the beginning of the storage period and each 7 days until 28 days of storage was complete. The fermented milks were stored at 4 °C in a climatic chamber (Model MA 415, Marconi, Piracicaba, Brazil).

#### 2.7.1. Determination of Viability

The viability of the probiotic culture was determined by the *Spread plate* technique on MRS agar medium. The fermented milk samples were subjected to several serial dilutions in 0.85 g/100 g saline solution. The selected dilutions were plated on MRS agar and immediately incubated at 37 °C for 48 h. After the incubation period, the number of Colony Forming Units (CFU) was determined. The final result was expressed as log CFU/mL.

#### 2.7.2. Technological Properties of Fermented Milks

For the technological evaluation of the fermented milks during the refrigerated storage, the pH, the titratable acidity and the color were evaluated. The pH and the titratable acidity were determined as described in Section 2.3.

The evaluation of the color of the fermented milks was made by using the ColorQuest XE Spectrophotometer (HunterLab, Reston, VA, USA), obtaining the values of the coordinates *L*, *a* and *b* of the Hunter system. From the values of the coordinates, *L*, *a* and *b*, the parameters related the hue angle (*h*, Equation (1)), the chroma (*C*, Equation (2)) and the color difference (Δ*E*, Equation (3)) were obtained [17,18,19].

(1) h = arctang(b/a)

(2) $$C=\sqrt{\left(a^{2}+b^{2}\right)}$$

(3) $$\Delta E=\sqrt{{\left(L-L_{0}\right)}^{2}+{\left(a-a_{0}\right)}^{2}+{\left(b-b_{0}\right)}^{2}}$$

In which

*L*—measurable in terms of white to black intensity;
*a*—measurable in terms of red and green intensity;
*b*—measurable in terms of yellow and blue intensity;
*L*_0_, *a*_0_ and *b*_0_—coordinates obtained at the beginning of the storage period of each of the fermented milks.

### 2.8. Statistical Analysis

Initially, we performed an analysis of variance with 5% probability and later we performed Tukey’s test or a regression analysis. Post-hoc Tukey’s test and contrasts were used for the comparison of the variables related to the chemical composition and sensorial quality of fermented milks, respectively. For the viability of probiotics in fermented milks with different concentrations of GBP during refrigerated storage post-hoc analysis was done with Tukey’s test and Bonferroni’s correction. For the results related to the kinetic parameters during the fermentation process, syneresis and technological properties during the refrigerated storage, regression analysis was used. The analysis of variance and Tukey’s tests were performed in SPSS v. 19.0 (IBM Corporation, New York, NY, USA). SigmaPlot software v.10 (Systat Software Inc., Erkrath, Germany) was used. In each experiment, three replicates were adopted, except for the sensory analysis which was evaluated with 113 individuals.

## 3. Results and Discussion

### 3.1. Kinetic Parameters of Acidification

**Figure 1:**
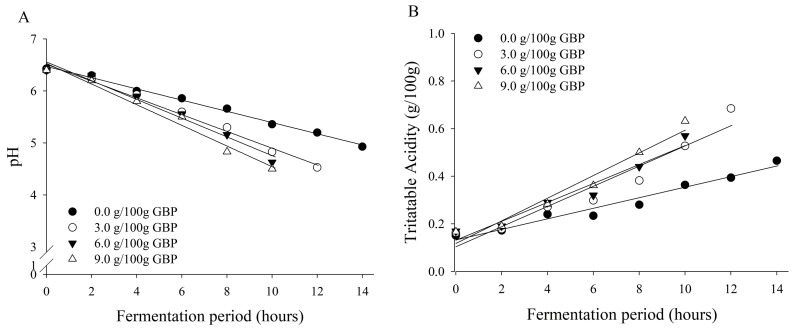
Regression curves of pH (**A**) and titratable acidity, expressed in g/100 g of lactic acid (**B**) of fermented milk by *L. paracasei* subsp *paracasei* LBC 81 with different concentrations (g/100 g) of green banana pulp (GBP) as a function of the fermentation period.

The pH and titratable acidity profile of the formulations of milks fermented by *L. paracasei* LBC 81 can be observed in Figure 1 and Table 1.

It was verified that as the concentration of GBP increased in the formulations, the decrease in the pH was faster and hence, there was a faster increase in titratable acidity. In regard to the pH variable (Figure 1A), estimated values equivalent to 5.37 and 4.53 after 10 h of fermentation were obtained for the control treatment control (0.0 g/100 g) and the treatment with 9.0 g/100 g of GBP, respectively. In relation to the titratable acidity (Figure 1B), the estimated values for the control treatment (0.0 g/100 g) and with 9.0 g/100 g of GBP were equivalent to 0.33 and 0.61 g/100 g of lactic acid, respectively.

Results referring to the fermentation kinetics demonstrated that GBP is an excellent substrate for the growth of *L. paracasei* LBC 81. GBP presents a great source of resistant starch (RS), phenolic acids, minerals and vitamins of importance to the human health [12] and, probably to the growth of *L. paracasei* LBC 81. Although the milk enriched with GBP was subjected to the autoclaving process, which reduces the presence of some thermosensitive nutrients, the amount of nutrients that remained was enough to accelerate the acidification of the product by the microorganism fermentation. Probably, the incorporation of GBP into the fermented milk allowed the prevalence of the homofermentative metabolism of the *L. paracasei* subsp *paracasei* LBC 81 culture. The addition of GBP stimulated the growth of *Lactobacillus acidophilus* after one day of fermentation in research carried out by Costa et al. [10]. 

*L. paracasei* strains present an optional heterofermentative metabolism, which makes them undesirable in some situations for use as starter cultures, being more used in the elaboration of dairy products as an adjunct culture. In anaerobic and nutrient-restricted conditions, the homofermentative pathway may undergo changes due to the activity of the NAD-dependent pyruvate decarboxylase enzyme, generating acetate and CO_2_ from pyruvate, and thereby displacing the production of lactic acid [20].

Probiotic bacteria, when utilized as starter culture, are responsible for the slower acidification of a product, by a duration of almost 38 h [21]. Therefore, the elaboration of probiotic yoghurt has been one alternative used. The symbiotic association of the yogurt cultures (*Lactobacillus delbrueckii* and *Streptococcus thermophilus*) with probiotic strains stimulates the growth of other probiotic cultures. However, the elaboration of fermented milk using only one probiotic culture as a starter has been possible because this incorporates foods rich in nutrients which benefits the growth of the microorganism. The fermentation of the milk by probiotic strains (*L. rhamnosus* IMC 501^®^ e *L. paracasei* IMC 502^®^) presented a higher decrease in pH when the milk was supplemented with wheat and oat bran [22].

### 3.2. Chemical Composition

There were significant differences (*p* < 0.05) only in the variables moisture and crude fiber (Table 2).

The moisture obtained in the fermented milk enriched with 9.0 g/100 g of GBP was significantly lower (*p* < 0.05) than that obtained in the product with 3.0 g/100 g of GBP and the product without GBP. This result was expected, because when the total amount of GBP is higher, the percentage of dry matter increases and consequently, the amount of moisture reduces.

GBP samples presented more crude fiber (*p* < 0.05) than the control (0.0 g/100 g) sample due to the amount of fiber content present in green banana pulp [12,23]. The control sample (0.0 g/100 g) does not include any ingredient that contains fiber.

Dietary fiber has been widely associated with positive health outcomes (satiety, glycemic index regulation, intestinal regulation, cancer prevention and others) with the fiber content of food products being a potential basis for health claims in several countries [24]. Therefore, our product could have positive effects on human health, not only due to its probiotic effect, but also due to the effect of fiber on human organisms. In addition to the fiber content, GBP can also contribute to human health (as glycemic and cholesterol control, intestinal regulation, chronic disease prevention, and satiety) due to the presence of resistant starch and phenolic compounds [25,26]. Since we did not evaluate these compounds, further studies are necessary to evaluate the presence of these compounds and the effect of GBP fermented milk in human health.

It is important to highlight that the addition of GBP on these concentrations (3.0–9.0 g/100 g) did not significantly affect the composition of total protein content, lipids and carbohydrates. Therefore, there is probably no interference on total energetic value from the final formulation.

### 3.3. Sensory Properties

In all evaluated attributes, a significant difference (*p* < 0.05) was observed (Table 3).

The fermented milk enriched with 6.0 g/100 g of GBP presented the highest mean acceptance regarding appearance and texture (*p* < 0.05). There was no significant difference (*p* > 0.05) when comparing fermented milk with 6.0 g/100 g of GBP with fermented milk with 3.0 g/100 g of GBP for flavor, aroma and overall acceptability. However, there was a significant difference (*p* < 0.05) when fermented milk without GBP (0.0 g/100 g) was compared with fermented milks with 3.0 and 6.0 g/100 g of GBP for flavor and overall acceptability.

It is important to highlight that 70.0% or more of the panelists expressed mean values ranging from 6–9 (on 9-point hedonic scale) for fermented milk with 6.0 g/100 g of GBP for all evaluated attributes. A sample is considered as having good acceptance when 70% or more of the individuals express mean values on the 9-point hedonic scale of higher than 5 [27]. Similarly, a study that evaluated the acceptance of yoghurt with GBP showed better acceptance for all evaluated attributes with the incorporation of GBP at a concentration of 5.0 g/100 g in yoghurt [11]. In relation to fermented milk with 3.0 g/100 g, the percentage of panelists expressing mean values of 6–9 on the 9-point hedonic scale was also 70.0% or more, except for texture. The difference in the texture is related to the addition of GBP which, among other functions, is employed to change the consistency of liquid products [24].

It is noted that the formulation with 9.0 g/100 g of GBP was less accepted regarding all attributes evaluated, compared to other formulations with GBP. Considering that the chemical composition of formulations with 6.0 and 9.0 g/100 g of GBP did not differ statistically, the use of the formulation with better acceptance (6.0 g/100 g of GBP) would probably not negatively affect the nutritional impact of this product. The concentration of 9.0 g/100 g of GBP negatively affected the acceptance of the product probably due to the acidification of the product caused by the increase in the concentration of GBP (Figure 1 and Table 1). Thus, the rejection observed related to the attributes, flavor and overall quality, can be associated with the higher acidity of the 9.0 g/100 g GBP product, since flavor impacts directly on the overall quality of the product. Moreover, the reduction in humidity and the increase in the solid content could be affected the texture of the 9.0 g/100 g GBP product.

### 3.4. Syneresis in the Fermented Milks

**Figure 2:**
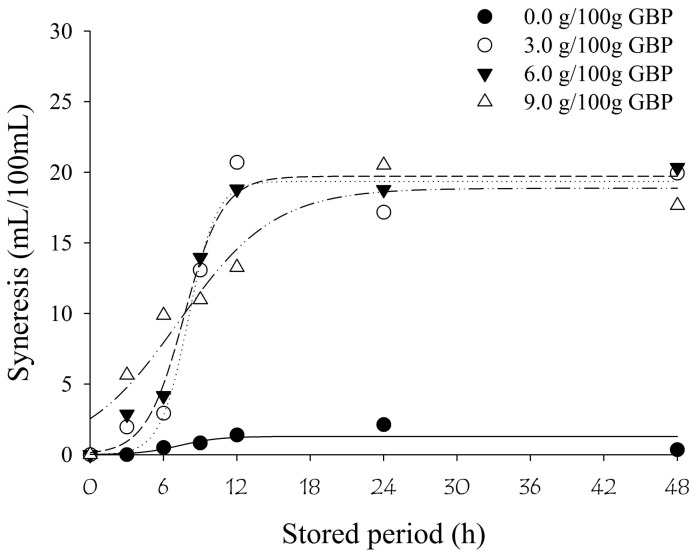
Regression curves of syneresis (g/100 g) of milk fermented by *L. paracasei* subsp *paracasei* LBC 81 with different concentrations of green banana pulp (GBP) over 48 h at 4 °C.

Regarding syneresis in the fermented milks for up to 48 h at 4 °C (Figure 2 and Table 4), it was observed that the fermented milk without GBP presented lower syneresis compared with fermented milks enriched with GBP.

When the samples containing GBP were analyzed, faster syneresis was observed until 6 h of storage in the fermented milk with 9.0 g/100 g of GBP. In contrast, it was verified that there was a tendency toward higher syneresis in fermented milks containing 3.0 and 6.0 g/100 g of GBP until 24 h of storage. It should be highlighted that during the evaluation of the syneresis in the fermented milk with 9.0 g/100 g of GBP, a physical retention of the serum by the gel was observed, and it was not possible to collect it through the top of the bottles used to pack the product.

Due to its high content of starch, green banana pulp, [28], was probably responsible for enhancing the syneresis of the fermented milks [29]. Syneresis occurred due to the high tendency for hydrogen bonds to form between adjacent molecules of starch, when refrigerated. This process is known as retrogradation and results in the appearance of gel [30]. Over time, this formed gel has the tendency to release water, known as starch syneresis.

It is important to highlight that in dairy products, such as yoghurts and fermented milks, regardless of the presence of starch, syneresis can be observed during refrigerated storage due to the acidity and changes in protein binding [24]. Therefore, the addition of GBP that contains native and resistant starch accentuates the syneresis of our product.

### 3.5. Viability of L. paracasei LBC 81 during Storage

The mean values for the viability of *L. paracasei* LBC 81 in fermented milks made with different amounts of GBP for 28 days of storage at 4 °C are presented in Table 5.

Significant variation (*p* < 0.05) in the viability of *L. paracasei* LBC 81 was observed based on the interaction between the GBP concentration and storage period. During the storage period, only fermented milk enriched with 3.0 g/100 g of GBP showed no significant variation (*p* > 0.05) in the population size of *L. paracasei* LBC81. It should be noted that fermented milks with 6.0 and 9.0 g/100 g of GBP had higher counts of 9.0 log CFU/g after 28 days of storage at 4 °C.

Variations in the population size of the microorganisms, similar to that observed for *L. paracasei* LBC81 in fermented milks, are expected since microorganisms present in food stored under refrigeration undergo several physiological changes, which may lead to a decrease or increase in the microbial population. In addition, the decrease or increase in the population of *L. paracasei* LBC81 did not reach a logarithmic cycle. Although this variation was significant, it should be noted that all formulations developed were within the established standard for probiotic foods (10^6^ to 10^9^ CFU/g) [31].

When analyzing the effect of the addition of GBP on the viability of *L. paracasei* LBC 81 in each storage period, a significant variation (*p* < 0.05) was observed. In general, there was an increase in the population of *L. paracasei* LBC81, associated with the elevation in GBP concentration. Green banana is rich in various nutrients, and the availability of these nutrients in fermented milk may have favored the growth of *L. paracasei* LBC81 during the storage period. Stability or population increase of probiotic strains during the storage period is desirable when associated with the ingestion of a larger number of probiotic strains. On the other hand, it becomes undesirable because it is associated with post-acidification, which can make the product very acidic and of low acceptance by the consumer.

The results obtained are similar to those observed in fermented milks elaborated with fiber from oranges or quinoa [8,32]. However, previous studies have shown that supplementation does not always maintain the stability of a probiotic culture throughout the storage period. Supplementation of yogurt with fruit flours, such as banana, apple and grape, was not sufficient to maintain stability for 28 days of storage at 4 °C [9]. However, the reduction in viability was less than one log cycle, without compromising the functionality of the product.

### 3.6. Technological Properties

**Figure 3:**
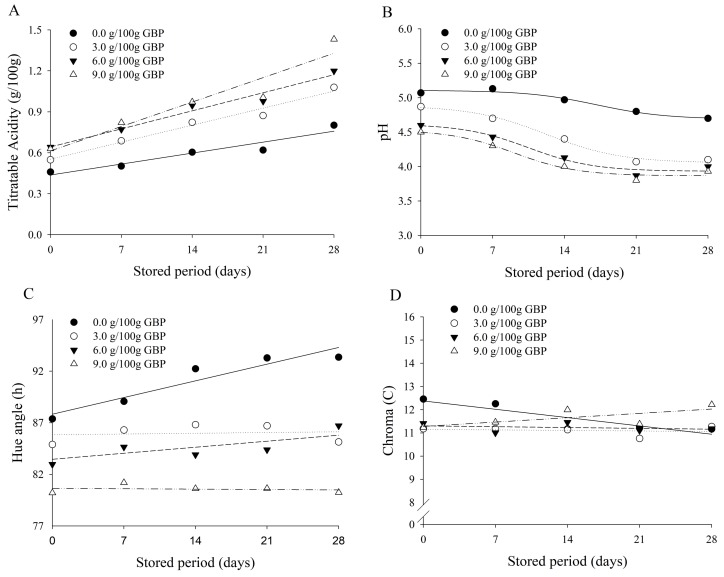

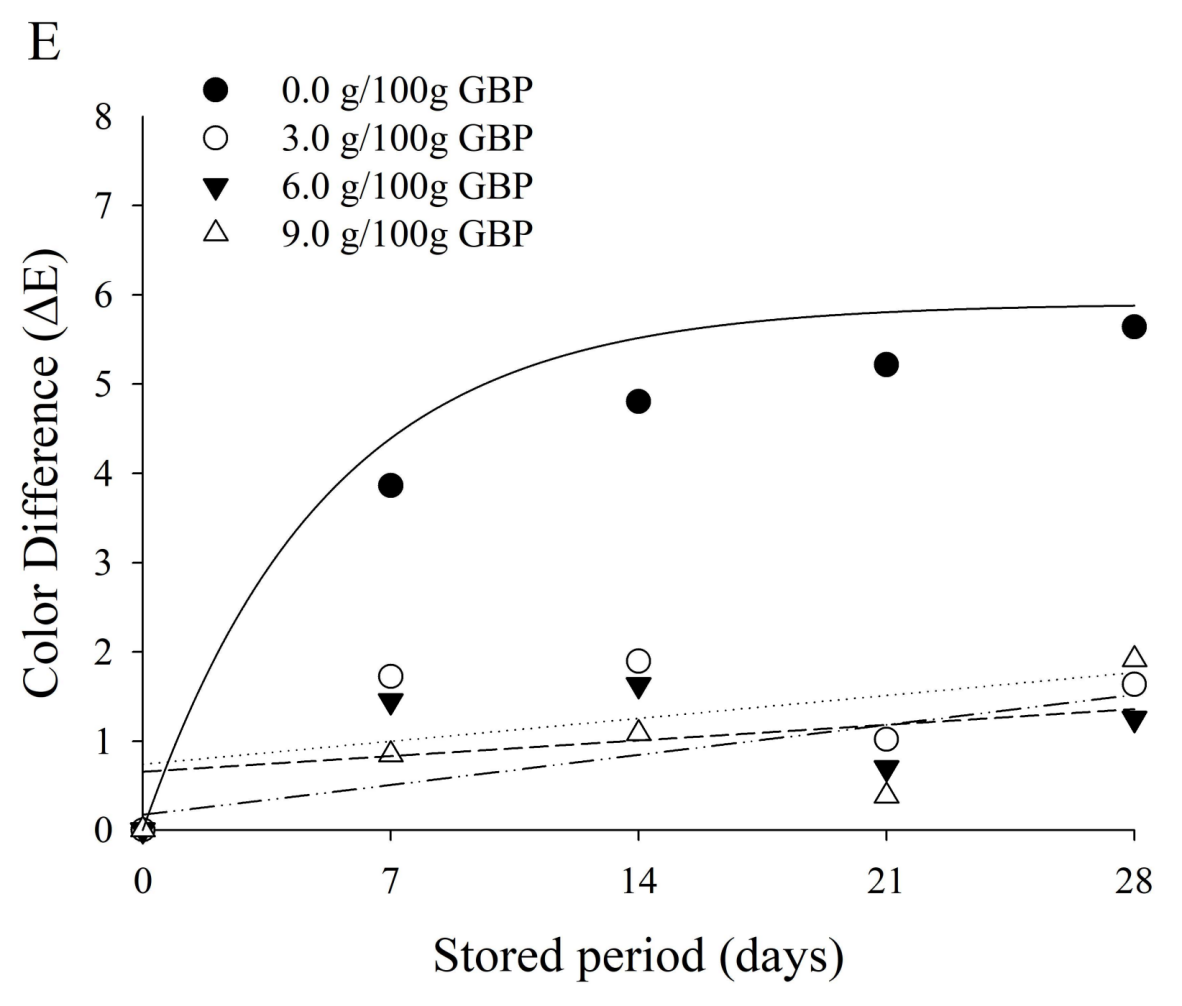
Regression curves of the titratable acidity expressed in g/100 g of lactic acid (**A**); pH (**B**); hue angle (**C**); Chroma (**D**) and Color Difference (Δ*E*) (**E**) of fermented milks by *L. paracasei* subsp *paracasei* LBC 81 with different concentrations (g/100 g) of green banana pulp (GBP) as a function of the fermentation period.

The technological properties of the fermented milk elaborated with different concentrations of GBP over 28 days of storage at 4 °C are presented in Figure 3 and Table 6.

In relation to the titratable acidity (Figure 2A), it was observed that there was a significant increase as a result of the interaction between the concentrations of GBP and the storage period.

It should be noted that fermented milk containing GBP had higher initial titratable acidity than fermented milk without GBP, and there was a trend for this difference to increase throughout the storage period. At the beginning of the storage period, the estimated difference in titratable acidity between the fermented milk without GBP and that with 9.0 g/100 g of GBP was 0.17 g/100 g of lactic acid. However, after 28 days of storage, the estimated difference was 0.73 g/100 g of lactic acid. Such a trend may be associated with the greater viability of *L. paracasei* LBC 81 in fermented milks containing GBP, as observed in Table 5.

Regarding the pH data (Figure 3B), a consistency was observed with the titratable acidity data, since there was a reduction in pH as the storage period increased, with an increase in the difference between values observed in the fermented milks without GBP and those with GBP. The differences between pH values in fermented milks without GBP and with 9.0 g/100 g of GBP were 0.64 and 0.83 at the beginning of the storage period and after 28 days, respectively.

The stability of the pH and acidity of fermented dairy products during refrigerated storage is desirable with respect to the acceptability and viability of the probiotic culture. In the present study, we verified that an increase in the concentration of GBP in fermented milk caused a greater decrease in pH, and consequently elevated the acidity of the product. Similarly, a decrease in pH was also observed in probiotic yogurt supplemented with lemon and orange fibers after 30 days of storage at 4 °C, where the pH of yogurts supplemented with lemon and orange fiber were 3.97 and 3.92, respectively [32].

The post-acidity in the different formulations of the fermented milks enriched with GBP over the storage period of 28 days at 4 °C is not desirable. The yoghurts and fermented milks present a shelf life of 28 to 30 days. This established shelf life is associated with the pH and acidity of the product. These parameters, after a prolonged storage period, make the products less acceptable for consumers. In our study, we observed that the product with higher acidity was less accepted by consumers. In addition to compromising product acceptance, the marked decrease in pH may affect the viability of probiotic strains. It is important to highlight that even with the decrease in pH and the increase in the titratable acidity of the fermented milks enriched with GBP, the viability of *L. paracasei* LBC 81 was not compromised over 28 days of storage at 4 °C (Table 5).

As for the coloration of fermented milks during storage, there was a significant variation in the variables, hue angle (*h*), chroma (*C*) and color difference (Δ*E*), due to the interaction between the concentration of GBP and the storage period. At the beginning of the storage period, the hue angle (Figure 3C) of the fermented milk without GBP was higher than in the fermented milks containing GBP. As the storage period increased, there was only a significant (*p* < 0.05) increase in the hue angle in fermented milk that did not contain GBP. Similar results were observed for the chroma (Figure 3D) and for the color difference (Figure 3E). In terms of chroma, there was a significant decrease (*p* < 0.05) only in the product without GBP over 28 days of storage. In the same sense, an expressive variation was observed only in the product elaborated without GBP, with an estimated value of 5.88, after 28 days of storage. On the other hand, the color differences in the fermented milks with GBP remained below 2.00 during storage at 4 °C.

In general, foods are susceptible to oxidation reactions when submitted to some storage conditions. Those foods rich in antioxidant substances are protected by the damage caused from the oxygen reactions, and therefore, present less alteration to flavor and color. Possibly, substances present in banana, such as antioxidants and phenolic compounds [33], acted as protective agents in the fermented milks against the action of reactive oxygen species. In this sense, these substances influenced the stability of the hue angle, chroma and color difference of fermented milk over 28 days of storage at 4 °C.

Although antioxidants and phenolic compounds are sensitive to high temperatures, it is possible that, after autoclaving, reduced concentrations of these substances were sufficient to ensure the color stability of the fermented milks. This stability of the color on the fermented milks with GBP can also justify the stability of the probiotic strain *L. paracasei* LBC 81 over the 28 days of storage at 4 °C. In the presence of antioxidant substances and phenolic compounds, *L. paracasei* LBC 81 would be least exposed to attack by reactive oxygen species. The oxygen reactive species attack proteins, nucleic acids and lipids and are considered one of the most important causes of injury and cellular death [34]. Thus, it is possible that the stability of the viability of *L. paracasei* LBC 81 is also associated with the protection of antioxidant substances and phenolic compounds.

## 4. Conclusions

The addition of GBP accelerated the growth and stabilized the viability of *L. paracasei* LBC 81 during a period of refrigerated storage. In addition to this benefit, the tonality, chroma and color difference of the fermented milks were less affected as the amount of GBP increased. The sensorial analysis of fermented milk enriched with 6.0% of GBP had better acceptance when compared to fermented milk without GBP and with the other formulations. One of the disadvantages of the incorporation of GBP into fermented milk was in relation to the increase in syneresis and the occurrence of post-acidification during the storage period. However, these technological variables can be improved with the use of stabilizers and with greater control of the fermentation process in further studies. It is also important to highlight that the use of green banana pulp can contribute to the nutritional quality of the fermented milk due to its phenolic compounds, resistant starch, fibers and other components. Therefore, our product could impact consumer health due to its probiotic and prebiotic effects. However, further studies should be conducted to evaluate the impact of this product on consumers’ health.

## Figures and Tables

**Table 1:** Regression equations of pH and titratable acidity (g/100 g of lactic acid) of fermented milks by *L. paracasei* subsp. *paracasei* LBC 81 with different concentrations (g/100 g) of green banana pulp (GBP) as a function of the fermentation period.

Variable	GBP (g/100 g)	Adjusted Equation	*R*^2^	SEE
pH	0.0	$\hat{y}=6.47-0.11{\;}^{**}\;X$	0.99	0.04
	3.0	$\hat{y}=6.51-0.16^{\;**\;}X$	0.99	0.08
	6.0	$\hat{y}=6.56-0.18{\;}^{**\;}X$	0.97	0.13
	9.0	$\hat{y}=6.53-0.20{\;}^{**}\;X$	0.98	0.13
Titratable Acidity (g/100 g)	0.0	$\hat{y}=0.13+0.02{\;}^{**}\;X$	0.96	0.02
	3.0	$\hat{y}=0.10+0.04{\;}^{**}\;X$	0.92	0.06
	6.0	$\hat{y}=0.13+0.04{\;}^{**}\;X$	0.95	0.04
	9.0	$\hat{y}=0.11+0.05{\;}^{**\;}X$	0.96	0.04

SEE = Standard error of estimate; ** Significant (*p* < 0.01); *n* = 3 sample replicates.

**Table 2:** Chemical composition of fermented milks by *L. paracasei* subsp *paracasei* LBC 81 with different concentrations (g/100 g) of green banana pulp (GBP).

Variable	Concentration of GBP (g/100 g)			
	0.0	3.0	6.0	9.0
Moisture (g/100 g)	85.90 ± 0.40 ^a^	85.67 ± 0.41 ^a^	84.52 ± 1.39 ^a,b^	83.20 ± 0.74 ^b^
Proteins (g/100 g)	2.71 ± 0.14	2.84 ± 0.13	2.85 ± 0.76	3.34 ± 0.63
Lipids (g/100 g)	0.34 ± 0.10	0.37 ± 0.12	0.38 ± 0.12	0.21 ± 0.07
Ash (g/100 g)	0.88 ± 0.11	0.81 ± 0.10	0.85 ± 0.04	0.81 ± 0.10
Crude fiber (g/100 g)	0.00 ± 0.00 ^b^	0.40 ± 0.12 ^a^	0.43 ± 0.00 ^a^	0.48 ± 0.08 ^a^
Carbohydrates (g/100 g)	10.14 ± 0.57	9.92 ± 0.44	10.97 ± 1.61	11.96 ± 0.39

For moisture and crude fiber, means followed by the same lower-case letter ^(a–b)^ on the lines did not differ statistically with Tukey’s test (*p* > 0.05); *n* = 3 sample replicates.

**Table 3:** Sensory analysis of fermented milks by *L. paracasei* subsp *paracasei* LBC 81 with 0.0, 3.0, 6.0 and 9 g/100 g of green banana pulp (GBP).

Sensory Attribute	Concentration of GBP (g/100 g)			
	0.0	3.0	6.0	9.0
Appearance	5.13 ± 2.23 ^d^	7.56 ± 1.46 ^b^	8.12 ± 1.16 ^a^	5.68 ± 1.94 ^c^
Flavor	5.16 ± 2.32 ^b^	6.25 ± 1.76 ^a^	6.32 ± 1.89 ^a^	4.83 ± 2.35 ^b^
Aroma	6.48 ± 1.79 ^b,c^	6.90 ± 1.45 ^a,b^	7.00 ± 1.56 ^a^	6.15 ± 2.00 ^c^
Texture	4.98 ± 2.12 ^c^	6.28 ± 1.90 ^b^	7.05 ± 1.85 ^a^	4.94 ± 2.23 ^c^
Overall acceptability	5.30 ± 1.94 ^b^	6.63 ± 1.48 ^a^	6.78 ± 1.73 ^a^	5.02 ± 2.10 ^b^

For each sensory attribute, means followed by the same lower-case letters ^(a–d)^ on the lines do not differ significantly in post-hoc analysis via contrasts (*p* > 0.05); *n* = 113 panelists.

**Table 4:** Syneresis regression equations of fermented milks by *L. paracasei* subsp *paracasei* LBC 81 with different concentrations of green banana pulp (GBP) over 48 h at 4 °C.

GBP (g/100 g)	Adjusted Equation	*R*^2^	SEE
0.0	$\hat{y}=\frac{1.29}{1\;+\;e^{-\left(\frac{X-7.28}{1.63}\right)}}$	0.55	0.64
3.0	$\hat{y}=\frac{19.35}{1\;+\;e^{-\left(\frac{X-8.04}{1.14}\right)}}$	0.98	1.74
6.0	$\hat{y}=\frac{19.71}{1\;+\;e^{-\left(\frac{X-7.70}{1.57}\right)}}$	0.99	1.21
9.0	$\hat{y}=\frac{18.87}{1\;+\;e^{-\left(\frac{X-7.24}{3.91}\right)}}$	0.94	2.12

SEE = Standard error of estimate; *n* = 3 sample replicates.

**Table 5:** Evaluation of *Lactobacillus paracasei* subsp *paracasei* LBC81 in fermented milks enriched with different concentrations of green banana pulp (GBP) during a storage period of 28 days at 4 °C.

Stored Period (Days)	GBP Concentration (g/100 g)			
	0.0	3.0	6.0	9.0
0	8.89 ± 0.17 ^a,A^	9.05 ± 0.15 ^a,A^	8.98 ± 0.36 ^a,B^	8.91 ± 0.27 ^a,C^
7	8.36 ± 0.19 ^c,B^	9.01 ± 0.30 ^b,A^	9.83 ± 0.14 ^a,A^	9.56 ± 0.19 ^a,A,B^
14	8.90 ± 0.21 ^b,A^	8.95 ± 0.20 ^b,A^	9.47 ± 0.18 ^a,A^	9.32 ± 0.12 ^a,b,B,C^
21	8.95 ± 0.17 ^c,A^	9.16 ± 0.54 ^b,c,A^	9.60 ± 0.27 ^a,b,A^	9.83 ± 0.30 ^a,A^
28	8.66 ± 0.12 ^b,A,B^	8.91 ± 0.70 ^b,A^	9.43 ± 0.14 ^a,A,B^	9.57± 0.32 ^a,A,B^

Means followed by the same lower-case letters on the lines ^(a–c)^ did not differ significantly in post-hoc analyses by Tukey’s tests (*p* > 0.05). Means followed by the same capital letters ^(A–C)^ in a column did not differ significantly by in post-hoc analyses by Bonferroni’s corrections (*p* > 0.05).

**Table 6:** Regression equations of titratable acidity expressed in g/100 g of lactic acid (A), pH (B), hue angle (C), Chroma (D) and Color Difference (Δ*E*) of fermented milks by *L. paracasei* subsp *paracasei* LBC 81 with different concentrations of GBP over 28 days at 4 °C.

Variable	GBP (g/100 g)	Adjusted Equation	*R*^2^	SEE
Titratable Acidity (g/100 g)	0.0	$\hat{y}=0.44+0.01^{\;*\;}X$	0.91	0.05
	3.0	$\hat{y}=0.55+0.02^{\;**}\;X$	0.97	0.04
	6.0	$\hat{y}=0.64+0.02{\;}^{**}\;X$	0.97	0.05
	9.0	$\hat{y}=0.61+0.03{\;}^{*}\;X$	0.91	0.11
pH	0.0	$\hat{y}=4.69+\frac{0.41}{1\;+\;e^{-\left(\frac{X-17.12}{-3.36}\right)}}$	0.97	0.06
	3.0	$\hat{y}=4.05+\frac{0.83}{1\;+\;e^{-\left(\frac{X-12.23}{-3.63}\right)}}$	0.99	0.08
	6.0	$\hat{y}=3.93+\frac{0.69}{1\;+\;e^{-\left(\frac{X-10.48}{-3.17}\right)}}$	0.97	0.12
	9.0	$\hat{y}=3.87+\frac{0.66}{1\;+\;e^{-\left(\frac{X-9.11}{-3.87}\right)}}$	0.97	0.10
Hue angle	0.0	$\hat{y}=87.83+0.23{\;}^{*}\;X$	0.89	0.99
	3.0	$\hat{y}=85.79+0.01{\;}^{\mathrm{ns}}\;X$	0.02	1.02
	6.0	$\hat{y}=83.08+0.10{\;}^{\mathrm{ns}\;}X$	0.68	0.89
	9.0	$\hat{y}=80.67-0.01{\;}^{\mathrm{ns}}\;X$	0.04	0.45
Chroma	0.0	$\hat{y}=12.37-0.05^{\;*}\;X$	0.78	0.34
	3.0	$\hat{y}=11.16-0.01{\;}^{\mathrm{ns}}\;X$	0.04	0.23
	6.0	$\hat{y}=11.30-0.01{\;}^{\mathrm{ns}}\;X$	0.08	0.21
	9.0	$\hat{y}=11.28+0.03^{\;\mathrm{ns}}\;X$	0.48	0.35
Color Difference	0.0	$\hat{y}=5.90\left(1-e^{\left(-0.20X\right)}\right)$	0.91	0.88
	3.0	$\hat{y}=0.74+0.04^{\;\mathrm{ns}}\;X$	0.28	0.77
	6.0	$\hat{y}=0.66+0.3{\;}^{\mathrm{ns}}\;X$	0.18	0.70
	9.0	$\hat{y}=0.17+0.05^{\;\mathrm{ns}\;}X$	0.53	0.57

SEE = Standard error of estimate; ^ns^ Not significant; ** Significant (*p* < 0.01); * Significant (*p* < 0.05); *n* = 3 sample replicates.
